# Early and late effects of objecthood and spatial frequency on event-related potentials and gamma band activity

**DOI:** 10.1186/s12868-015-0144-8

**Published:** 2015-02-26

**Authors:** Matt Craddock, Jasna Martinovic, Matthias M Müller

**Affiliations:** Institute of Psychology, University of Leipzig, 04109 Leipzig, Germany; School of Psychology, University of Leeds, Leeds, LS9 2JT UK; School of Psychology, University of Aberdeen, Aberdeen, AB24 3FX UK

**Keywords:** Electroencephalography (EEG), Oscillations, Gamma band, Object recognition

## Abstract

**Background:**

The visual system may process spatial frequency information in a low-to-high, coarse-to-fine sequence. In particular, low and high spatial frequency information may be processed via different pathways during object recognition, with LSF information projected rapidly to frontal areas and HSF processed later in visual ventral areas. In an electroencephalographic study, we examined the time course of information processing for images filtered to contain different ranges of spatial frequencies. Participants viewed either high spatial frequency (HSF), low spatial frequency (LSF), or unfiltered, broadband (BB) images of objects or non-object textures, classifying them as showing either man-made or natural objects, or non-objects. Event-related potentials (ERPs) and evoked and total gamma band activity (eGBA and tGBA) recorded using the electroencephalogram were compared for object and non-object images across the different spatial frequency ranges.

**Results:**

The visual P1 showed independent modulations by object and spatial frequency, while for the N1 these factors interacted. The P1 showed more positive amplitudes for objects than non-objects, and more positive amplitudes for BB than for HSF images, which in turn evoked more positive amplitudes than LSF images. The peak-to-peak N1 showed that the N1 was much reduced for BB non-objects relative to all other images, while HSF and LSF non-objects still elicited as negative an N1 as objects. In contrast, eGBA was influenced by spatial frequency and not objecthood, while tGBA showed a stronger response to objects than non-objects.

**Conclusions:**

Different pathways are involved in the processing of low and high spatial frequencies during object recognition, as reflected in interactions between objecthood and spatial frequency in the visual N1 component. Total gamma band seems to be related to a late, probably high-level representational process.

## Background

The visual system parses an enormous amount of information in order to make sense of the world. Several models of the visual system describe a coarse-to-fine sequence of parsing of visual information imposed by differential processing of spatial frequencies [1-3]; specifically, low spatial frequencies (LSF) are privileged at early processing stages whereas high spatial frequencies (HSF) are privileged at later processing stages. Early, rapid processing of LSF via the magnocellular visual pathway and orbito-frontal cortex may have a critical role in object recognition: LSF provide information at a coarse scale, and since they vary slowly across space, they provide relatively clear information and a stable estimate of the global shape of an object or scene. LSF provide sufficient coarse information to allow a reasonable guess at the identity of an object and guide subsequent processing of HSF in ventral visual cortices [4]. The global precedence effect, in which the global shape of a hierarchical form is recognized faster than the local shapes which constitute it, may be underpinned by speeded processing of LSFs [5-7]. HSF vary much more rapidly across space, providing noisier, less stable information which is nevertheless more specific to the exact object or scene which is seen [8]. Thus, HSFs support finer analysis, such as the identification of the local shapes, or discriminations between subordinate object categories [9].

Event-related potentials (ERPs) are a valuable tool for examining the temporal evolution of neural activity, and thus provide a window into early interactions between objecthood and spatial frequency. Previous ERP experiments have examined the effects of differing spatial frequency bands in face and object recognition on the early perceptual P1 and N1 components, with somewhat mixed results. For example, Pourtois, Dan, Grandjean, Sander and Vuilleumier [10] found the P1 was enhanced for unfiltered faces relative to either high or low frequency filtered faces, and that the N170 was almost abolished for filtered faces. In contrast, Nakashima et al. [11] found a greater positivity in the P1 to LSF faces and a greater negativity in N170 to HSF faces. However, we also previously found a greater positivity in the P1 and greater negativity in the N1 for HSF relative to LSF objects using a categorization task [12]. However, we did not directly contrast responses to objects with responses to non-objects, and thus it is not clear whether the differences in responses reflect an object-specific method of processing stimuli or a more general property of the visual system.

Additionally, there are obvious parallels between early and late processing stages in models such as Bar et al.’s [4] and patterns of oscillatory activity in the gamma (>30 Hz) frequency band [13] typically observed during object recognition. Gamma band activity (GBA) may fulfil several critical roles in visual perception (see [14] for a recent review), and in the context of object recognition may reflect synchronization of disparate neural populations representing individual features of an object, and their binding into a unified percept. An early, evoked (time- and phase-locked) GBA (eGBA) signal is typically observed 50–150 ms after stimulus onset as a clear peak in the lower gamma frequency range (30–40 Hz). eGBA is highly sensitive to low-level stimulus properties such as complexity [15] and size [16], but generally does not differ between familiar and unfamiliar objects [15,17-20]. Fründ, Busch, Körner, Schadow, and Herrman [21] found a stronger early (60–120 ms) eGBA response and greater phase-locking to low frequency sine- or square-wave gratings (1 cycle per degree; cpd) than to high frequency gratings (10 cpd), and speculated that eGBA predominantly reflects magnocellular pathway excitation.

In contrast, *induced* – neither time- nor phase-locked – GBA (iGBA) is increased for familiar relative to unfamiliar objects in a window approximately 200–400 ms after stimulus onset ([15], [17], [19], e.g. [22]). Although iGBA findings might be compromised by a muscle artefact arising from miniature eye movements [23], correction methods for such artefacts now exist [24,25] and allow once again the examination of gamma band activity. Unlike eGBA, iGBA is relatively insensitive to low-level stimulus properties such as complexity [15]. Nevertheless, it may be sensitive to spatial frequency: Adjamian et al. [26] found that iGBA recorded using MEG peaked in response to stimuli with a spatial frequency between 2–4 cpd. Hadjipapas et al. [27] found much greater increases in iGBA for 3.5 or 6 cpd gratings than for 0.5 cpd gratings. However, the stimuli in both of these studies were sine- or square-wave gratings, and the suggested neural sources were in early visual cortices. It is not clear whether these findings would extend to GBA in relation to more complex stimuli or originating in higher-order visual cortices. The hypothesized representational role of the iGBA and its relatively late latency would suggest that it should be relatively unaffected by low-level factors such as spatial frequency.

An additional issue with many studies examining how spatial frequency is important in object recognition is that the specific cut-off thresholds vary widely, and are rarely motivated directly by physiological or experimental evidence. A recent fMRI study found that the orbito-frontal cortex/magnocellular route, which is critical to Bar et al.’s [4] model of object recognition, exhibits a greater response to stimuli with most energy between 0.17 and 0.38 cycles per degree of visual angle. In contrast, ventral areas critical to object recognition more strongly respond to stimuli which are chiefly composed of energy above 4.76 cpd [28]. We chose, therefore, to contrast responses to images of objects and non-objects which were either unfiltered (i.e. contained the full range of spatial frequency information) or filtered to remove selected frequency ranges (i.e. HSF or LSF only images) in line with those which evoked the strongest responses in the relevant cortical areas [28]. Given that the P1 is typically associated with sensitivity to low-level stimulus characteristics, we expected to see only effects of spatial frequency in the P1, without effects of objecthood. In contrast, higher-level characteristics (i.e. the presence or absence of an object) may be reflected in the N1, and it is in the N1 we might expect to see interactions between spatial frequency and objecthood. We expected that spatial frequency would modulate eGBA, but that objecthood would not. In contrast, the later, induced GBA response should be highest in response to objects but should not be modulated by spatial frequency. Note that here we examine total GBA (tGBA), which includes both iGBA and eGBA. A common earlier practice was to subtract the ERP from each trial before transformation to the time-frequency domain in order to separate the evoked from the induced gamma. However, this procedure has little practical impact on the analysis of the late, non-phase locked gamma response. Furthermore, it assumes that the evoked response is stationary, and may introduce unwanted frequencies into the supposedly “induced” response [29].

## Methods

### Participants

We recruited fifteen participants (ages 19 – 32, mean = 24 years) from the participant database of the University of Leipzig EEG-Laboratory. Thirteen were right-handed, 2 left-handed. Three were male, 12 female. The study conformed to the Code of Ethics of the World Medical Association and was approved by the local ethics committee of the University of Leipzig. Individual written informed consent was obtained. Subjects either received class credit or a small honorarium for participating.

### Stimuli and apparatus

**Figure 1:**
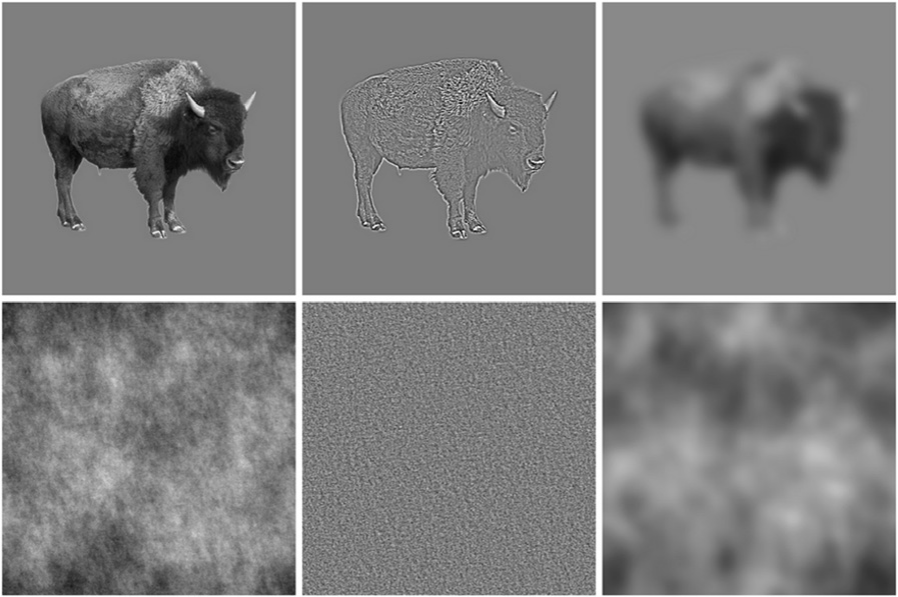
**Sample stimuli.** Columns show unfiltered, high-pass filtered, and low-pass filtered images. Noise images in the lower row were created by randomizing the phase of the FFT of the intact object. All pictures were matched for global luminance and RMS contrast.

Two-hundred and forty grayscale photographs depicting a single object against a neutral grey background were drawn from a commercial image database (Hemera Photo Objects). One-hundred and twenty of these photographs were of man-made objects, such as items of furniture; the other 120 were of natural objects, such as animals or fruit. Each stimulus (including the surrounding grey background) was 400 × 400 pixels. To create HSF and LSF versions of each object, we first converted each broadband image to the frequency domain using the fast Fourier transform. We then multiplied the amplitude of the frequency spectrum with a Gaussian low-pass filter with a cut-off at ~4.7 cycles per degree for HSF images or high-pass filter with a cut-off at ~0.9 cpd for LSF images. To produce non-object noise textures corresponding to the HSF, BB, and LSF images, we randomized the phase of the frequency spectrum of each unfiltered and filtered image. Thus, each noise texture had the identical amplitude spectrum and spatial frequency content as the image from which it was derived. We also matched the mean (global luminance) and standard deviation (RMS contrast) of every image to the mean global luminance and RMS contrast of the full set of BB images. Participants viewed the stimuli on a 17″ monitor (refresh rate 85 Hz) at a screen resolution of 1024 × 768 pixels from a distance of 80 cm. At this distance, the stimuli subtended approximately 10 degrees of visual angle in each direction (Figure 1).

### Design

We manipulated two factors: Object (object or non-object noise texture) × Spatial Frequency (HSF, BB, LSF). There were 480 trials in total, with 80 trials per condition. In object conditions, half of the trials showed natural objects, while half showed man-made objects. The objects presented in each condition were counterbalanced across participants, such that each object was presented an equal number of times in every condition once data from all participants was collected. The order of presentation of the stimuli was randomized for each participant. Trials were split into six blocks of 80, to allow brief rest periods for participants during the EEG recording.

Trials began with a white fixation cross on a black background presented for a randomly varying period of 500–800 ms. The fixation cross was then removed and a stimulus image presented for 500 ms, after which the image disappeared and was replaced with a fixation cross for 1000 ms. The screen was then blank for a variable period of 900–1200 ms. Participants were encouraged to use this time to blink, and avoid making eye movements or blinks while a stimulus or fixation cross was visible on screen. To respond, participants pressed different buttons to indicate whether each image showed a natural object, a man-made object, or a non-object texture. For analysis, we collapsed responses across man-made and natural objects, since our primary interest was in the contrast between objects and non-objects. Participants were given a practice block of 54 trials, in which we presented images created in the same way as those used in the actual experiment but drawn from a different set of objects. The Cogent toolbox for Matlab (Cogent, www.vislab.ucl.ac.uk/Cogent/; The Mathworks, Inc, Natick, Massachusetts) controlled the presentation of the stimuli.

### Behavioural data analysis

Reaction times and errors were analysed using a two-way repeated measures ANOVA with the factors Object (Object, Non-object) and Spatial Frequency (HSF, BB, LSF). Only reaction times on correct trials were included in the analysis. As noted above, the data were collapsed across living and non-living objects, since our primary interest was in the contrast between objects and non-objects. Note that for our error analysis, errors included categorizing a living object as a non-living object and vice versa, any object as a non-object, and any non-object as an object. Additionally, trials on which responses were not made were also counted as errors. Where necessary, Greenhouse-Geisser correction was used in cases of violations of sphericity, and significant effects were examined using post-hoc t-tests with Bonferroni-Holm correction for multiple comparisons. Generalized eta-squared is reported as a measure of effect size [30].

### EEG recording and analysis

We recorded continuous EEG from 64 locations at a sampling rate of 512 Hz using active Ag-AgCl electrodes connected to a BioSemi Active-Two amplifier system (BioSemi, Amsterdam, The Netherlands). Whereas many EEG amplifiers have a separate, “ground” electrode, the BioSemi system has two active electrodes placed close to electrode POz of the international 10–20 system [31]: Common Mode Sense (CMS) acts as a recording reference and Driven Right Leg (DRL) serves as ground [32,33]. We also used four electrooculograms (EOG) – above and below the right eye and outer canthi of each eye – in order to exclude artefacts related to blinks and eye movements. Initial EEG data processing was performed using the EEGLAB [34] toolbox with in-house procedures running under the Matlab environment (The Mathworks, Inc, Natick, Massachusetts). The Fully Automated Statistical Thresholding for EEG Artifact Rejection (FASTER) EEGLAB plug-in was used to reject artefacts and interpolation of globally and locally artefact contaminated channels [35]. We corrected for miniature saccade artefacts following Keren et al.’s [25] approach, using the microDetect EEGLAB plug-in (https://github.com/craddm/microDetect).

#### ERP analysis

A 40 Hz low-pass Butterworth filter was applied to the data before conducting event-related potential (ERP) analyses. We assessed the P1 (85–130 ms) and N1 (145–220 ms) at two lateral occipital electrode pairs (P7/PO7 and P8/PO8) at which effects of object category are often displayed, typically with enhanced negativity for individual categories such as faces ([36], the N170, e.g. [37]) or cars [38]. We defined the clusters of electrodes and appropriate time windows for ERP analyses on the basis of visual inspection of grand mean plots. Note that since the studied components are well established, with typical time-windows and topographies, circularity in this selection is minimal [39]. We used the ERPLAB (http://erpinfo.org) plug-in for EEGLAB to calculate the mean amplitude and local peak latency for each component after subtracting the mean amplitude of a baseline window from 200 ms prior to stimulus onset until stimulus onset from each time point. To detect local peak latency, ERPLAB searched over the time periods specified above for the most positive (for the P1) or most negative (N1) amplitude which was not surpassed within ±9.8 ms (5 sampling points). This helps to prevent the detection algorithm from selecting values from the rising edge of a slope at the extremes of the time window. For example, if both the peak of the P1 and the beginning of the transition to a P2 are within the selected time range, then the peak of the P1 should be selected rather than the rising P2 slope. The absolute peak was taken if no local peak was found. The detected peaks were visually inspected for each participant to ensure that the appropriate peaks were captured. The P1 and N1 were analysed using a repeated-measures ANOVA with the factors Object (Object, Non-Object), Spatial Frequency (HSF, BB, and LSF) and Hemisphere (Left, Right). All trials were included in the analyses, including response errors; thus, as recommended by VanRullen [40], the data were not conditioned on participant’s behavioural performance. Where necessary, Greenhouse-Geisser correction was used in cases of violations of sphericity, and significant effects were examined using post-hoc t-tests with Bonferroni-Holm correction for multiple comparisons. Generalized eta-squared is reported as a measure of effect size [30].

#### Time-frequency analysis

Time-frequency representations were obtained using sliding-window FFT methods implemented in the FieldTrip toolbox [41]. We applied bandstop 2^nd^ order Butterworth filters from 84–86 Hz and from 49–51 Hz to remove activity relating to the (85 Hz) refresh rate of the monitor and 50 Hz power line noise respectively. The linear trend was also removed. Evoked power (time and phase-locked to stimulus onset) was estimated by first averaging the data across trials and then performing time-frequency transformations. Total power (both evoked activity and activity neither time- nor phase-locked to stimulus onset) was estimated by performing time-frequency transformations on each trial and then averaging across trials. High-frequency power (30 to 110 Hz in 4 Hz steps) was estimated using multitapers [42], with a fixed time window of 250 ms and 5 orthogonal Slepian tapers, yielding a frequency smoothing of approximately 12 Hz. All activity was normalized by dividing by the mean of a baseline window from 400 to 100 ms before stimulus onset, yielding a measure of percentage change relative to baseline activity.

Electrodes for the analysis of GBA were selected by averaging the data across all conditions and choosing the regions of maximal gamma band activity in parietal and occipital areas. Thus, an occipital cluster was selected for eGBA. The eGBA response has low variability between individuals, and typically peaks in the 30–40 Hz range approximately 100 ms after stimulus onset. Therefore, we examined power averaged across this frequency range and a time window from 50 to 150 ms after stimulus onset. eGBA was examined using a repeated measures ANOVA with the factors Object (Object or Non-Object) and Spatial Frequency (HSF, BB, or LSF).

For tGBA, we examined two bilaterally symmetric posterior clusters, which correspond to areas where tGBA is typically found after correction for miniature saccade artefacts [12,24,25]. We chose individual peak frequencies on the basis of grand mean activity averaged across all conditions for each individual participant, and analysed tGBA from 200 ms after stimulus onset until stimulus offset (500 ms), averaging across both the peak frequency and one frequency bin on each side of that frequency (i.e. for a peak frequency of 62 Hz, we averaged across the frequency range 58–66 Hz). tGBA was examined using a repeated measures ANOVA with the factors Object (Object or Non-Object) and Spatial Frequency (HSF, BB, or LSF).

## Results

### Behavioural data

Participants responded slower [*F*(1,14) = 80.38, *p* < .001, ƞ^2^_g_. = .28] and made more errors [*F*(1,14) = 62.27, *p* < .001, ƞ^2^_g_. = .53] when responding to objects (648 ms; 7% errors, of which 5% were incorrectly categorizing an object, 1% categorization of an object as a non-object, and 1% missed responses) than to non-objects (541 ms; 1%).

There were significant main effects of Frequency for RTs [*F*(2,28) = 92.08, *p* < .001, ƞ^2^_g_. = .06] and errors [*F*(2,28) = 68.59, *p* < .001, ƞ^2^_g_. = .5]. Participants responded fastest and most accurately to BB (575 ms, 2% errors) images, with slower but as accurate responses to HSF images (582 ms; 3%), and the slowest and least accurate responses to LSF images (627 ms, 8%).

**Figure 2:**
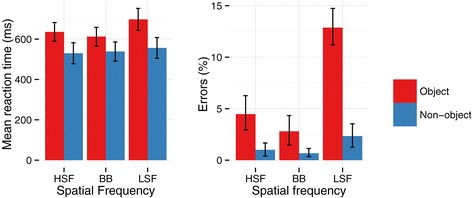
**Mean reaction times (ms) and errors (%).** Error bars indicate bootstrapped 95% confidence intervals.

There was a significant interaction between Object and Frequency for RTs [*F*(2,28) = 40.72, *p* < .001, ƞ^2^_g_. = .03] and errors [*F*(2,28) = 44.90, *p* < .001, ƞ^2^_g_. = .34], see Figure 2. Post-hoc tests indicated that RTs were significantly slower when an object was present than when there was no object on LSF (*p* < .001) and HSF trials (*p* = .02), while there were no significant differences between objects and non-objects on BB trials (*p* = .2). Additionally, responses to objects on LSF trials were slower than responses to both BB and HSF non-objects (both *p*s < .001), while responses on HSF object trials were slower than responses on BB (*p* = .04) non-object trials. There were significantly more errors committed for LSF objects than in any other condition (all *p*s < .001). There were also significantly more errors for HSF objects than for BB (*p* = .002) and HSF (*p* = .005) non-objects. There were no significant differences in errors between BB objects and non-objects (*p* = .2) or HSF objects and non-objects (*p*s = .4). Errors did not significantly differ across frequency for any non-objects (*p*s > .4).

### Event-related potentials

**Figure 3:**
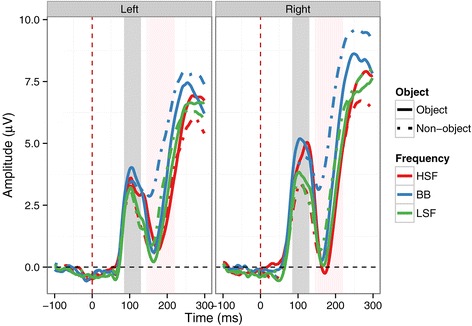
**ERP time course at the left and right parieto-occipital clusters. Solid lines show responses to objects, dashed lines responses to non-objects.** Red lines indicate responses to HSF images; blue lines indicate responses to BB images; green lines indicate responses to LSF images. Shaded grey area indicates P1 time window; shaded pink area indicates N1 time window.

**Figure 4:**
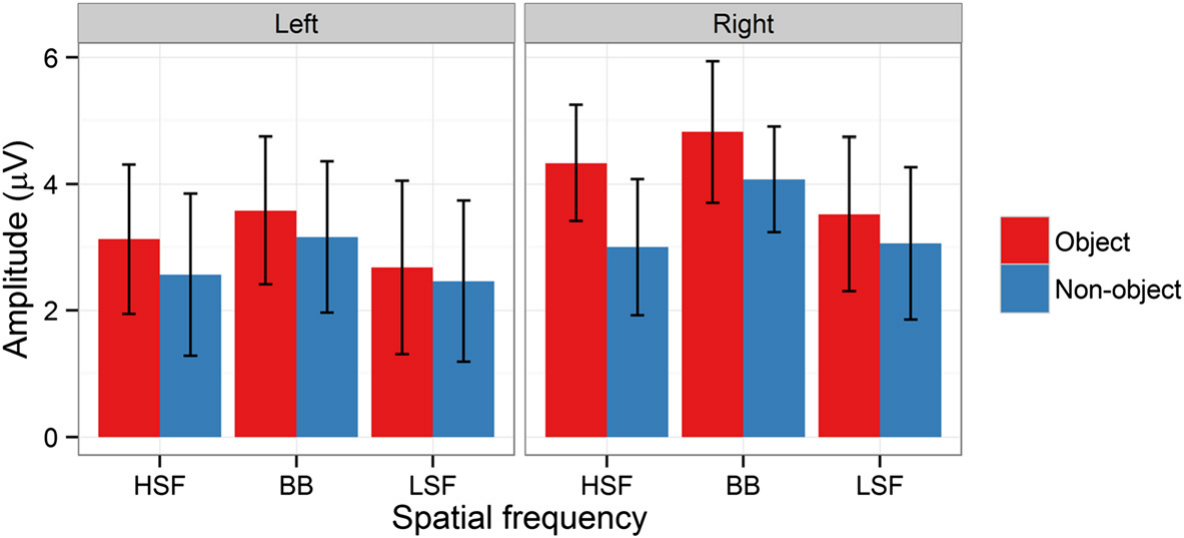
**The interaction between object and spatial frequency in P1 mean amplitudes for the Left and Right hemisphere electrode clusters.** Red bars show responses to objects, blue bars responses to non-objects. Error bars depict bootstrapped 95% confidence intervals.

In our analysis of P1 mean amplitudes, there was a significant main effect of Object [*F*(1,14) = 14.59, *p* = .002, ƞ^2^_g_. = .02], with more positive amplitudes on object (3.67 μV) relative to non-object (3.05 μV) trials. Furthermore, there was a significant main effect of Spatial Frequency [*F*(2,28) = 17.95, *p* < .001, ƞ^2^_g_. = .04]. Pairwise comparisons between each level of Spatial Frequency revealed that amplitudes were more positive for BB images (3.91 μV) than for HSF (3.26 μV; *p* < .001) and LSF images (2.93 μV; *p* < .001). Responses to HSF images were also significantly higher than to LSF images, despite the noticeably smaller difference (*p* = .01). Finally, there was a significant main effect of Hemisphere [*F*(1,14) = 6.54, *p* = .02, ƞ^2^_g_. = .04], with significantly more positive amplitudes in the right hemisphere (3.80 μV) than the left hemisphere (2.93 μV). No interactions were significant (all *p*s > .08). See Figures 3 and 4 for an overview. For P1 peak latency, no effects were significant (all *p*s > .08).

**Figure 5:**
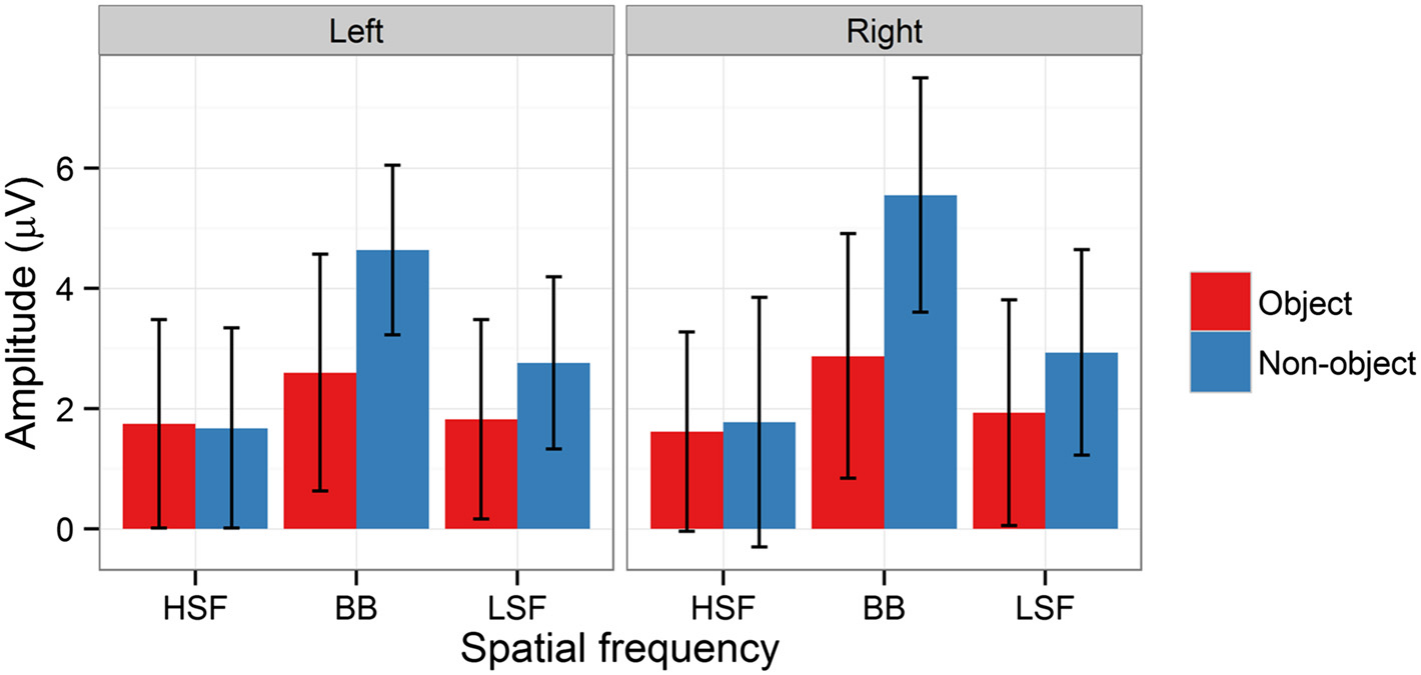
**N1 mean amplitudes for the interaction between object and spatial frequency at left and right hemisphere electrode clusters.** Error bars depict bootstrapped 95% confidence intervals.

In our analysis of N1 mean amplitudes, there was a significant main effect of Object [*F*(1,14) = 5.07, *p* = .04, ƞ^2^_g_. = .03], with a more negative N1 amplitude for objects (2.1 μV) than for non-objects (3.22 μV). There was also a significant main effect of Spatial Frequency [*F*(2,28) = 33.71, *p* < .001 ƞ^2^_g_. = .08], with more a negative N1 amplitude for HSF images (1.70 μV) than for BB (3.91 μV; *p* < .001) and LSF (2.36 μV; *p* = .01) images. LSF images were also more negative than BB images (*p* < .001). However, there was a significant interaction between Object and Spatial Frequency [*F*(2,28) = 6.08, *p* < .001, ƞ^2^_g_. = .02], see Figures 3 and 5. The N1 was significantly more negative for BB objects than for BB non-objects (*p* < .001), and more negative for LSF objects than for LSF non-objects (*p* = .03). However, there was no significant difference between HSF objects and HSF non-objects (*p* = 1). Thus, the N1 was sensitive to objecthood for BB and LSF but not HSF images. The N1 was also significantly more negative for HSF (*p* = .003) and LSF objects (*p* = .02) than for BB objects. Indeed, BB non-objects elicited significantly more positive amplitudes than any other combination of object and spatial frequency (all *p*s < .001). Neither the main effect of Hemsiphere nor any of the interactions involving Hemisphere were significant (all *p*s > .2).

For N1 latencies, there were significantly longer peak latencies [*F*(1,14) = 8.56, *p* = .01, ƞ^2^_g_. = .04] for objects (173 ms) than for non-objects (165 ms). There was also a significant main effect of Spatial Frequency [*F*(2,28) = 6.48, *p* = .005, ƞ^2^_g_. = .02], with significantly longer peak latencies for HSF images (173 ms) than for LSF images (166 ms). Peak latencies to BB images (168 ms) did not significantly differ from those for HSF or LSF images.

### Peak-to-peak analysis

In order to rule out the possibility that effects we observed in the N1 amplitude were carry-over effects from the P1, we also tested peak-to-peak amplitudes. We extracted the peak amplitude of each condition for the P1 and N1 and subtracted the N1 amplitude from the P1 amplitude, allowing us to quantify the N1 effects independently from the P1. We analysed peak-to-peak amplitudes with a repeated measures ANOVA with the factors Object (Object, Non-Object), Spatial Frequency (HSF, BB, LSF), and Hemisphere (Left, Right).

**Figure 6:**
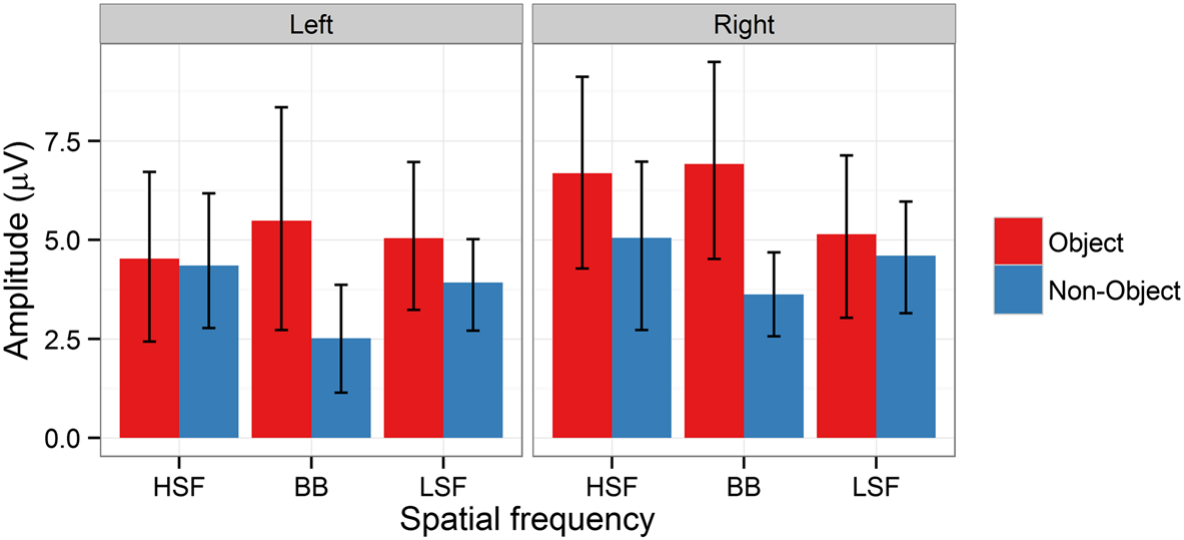
**Peak-to-peak (P1 minus N1) amplitudes for the interaction between object and spatial frequency at left and right hemisphere electrode clusters.** Error bars depict bootstrapped 95% confidence intervals.

There were significantly greater N1 amplitudes (i.e. a greater negative going deflection) for objects relative to non-objects [*F*(1,14) = 7.40, *p* = .02, ƞ^2^_g_. = .04]. There were no significant main effects of Spatial Frequency [*F*(2,28) = 2.33, *p* = .1, ƞ^2^_g_. = .003] or Hemisphere [*F*(1,14) = 1.81, *p* = .2, ƞ^2^_g_. = .02]. However, there were significant interactions between Object and Spatial Frequency [*F*(2,28) = 5.82, *p* = .003, ƞ^2^_g_. = .02] and Object, Spatial Frequency, and Hemisphere [*F*(2,28) = 3.68, *p* = .04, ƞ^2^_g_. = .003], see Figures 3 and 6. Significant two-way interactions should be considered cautiously when a three-way interaction is present. Nevertheless, in post-hoc tests the two-way interaction was driven by a significantly less negative N1 for BB non-objects than for BB (*p* = .005), LSF (*p* = .02) and HSF (*p* = .02) objects. Furthermore, it was less negative for BB non-objects than for LSF non-objects (*p* = .002), with marginal differences from HSF non-objects (*p* = .05). Other comparisons were not significant (*p*s > .2).

For the three-way interaction, no comparisons were significant (all *p*s > .07), rendering statistical decomposition of the interaction difficult. Inspection of Figures 3 and 6 suggests that the interaction is due to two small differences in the pattern across hemispheres. The difference between BB objects and non-objects is present in both left and right hemispheres. However, the smaller difference between HSF objects and non-objects present in the right hemisphere is absent from the left hemisphere. Similarly, a small difference between LSF objects and non-objects is apparent in the left hemisphere but absent from the right hemisphere.

### Evoked gamma band

**Figure 7:**
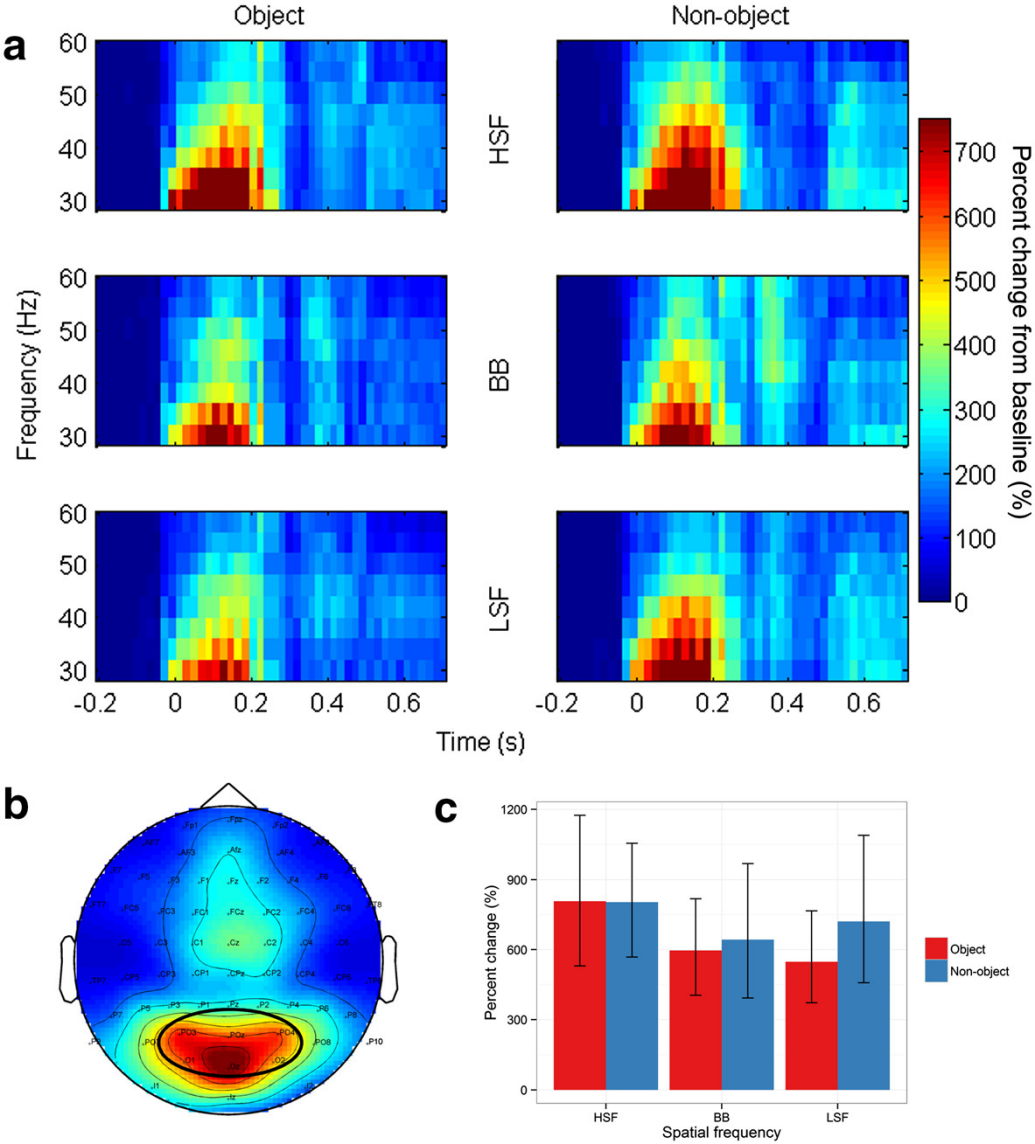
**Grand mean evoked GBA. (a) mean percent change from baseline in eGBA in the range 30 – 60 Hz, separately for each condition; left column shows activity on object trials, right column on non-object trials. (b)** Topographical representation of eGBA, averaged from 30–60 Hz and from 50-150 ms. Oval depicts electrode cluster selected for analysis. **(c)** bar graph depicting mean percent change from baseline of eGBA. Red bars show eGBA on object trials, blue on non-object trials. Error bars depict 95% confidence intervals.

As predicted, there was no effect of Object [*F*(1,14) = 1.30, *p* = .3, ƞ^2^_g_. = .005] on evoked gamma band activity. However, there was a significant effect of Spatial Frequency [*F*(2,28) = 5.29, *p* = .01, ƞ^2^_g_. = .02]. The change in evoked gamma band activity was significantly greater for HSF (805%) than BB (619%; *p* = .04) images. The difference between HSF and LSF images (634%) was marginal (*p* = .06), while the difference between LSF and BB images was clearly not significant (*p* = .8). Finally, the interaction between Object and Spatial Frequency was not significant [*F*(2,28) = 0.53, *p* = .6, ƞ^2^_g_. = .005], see Figure 7.

### Total gamma band

**Figure 8:**
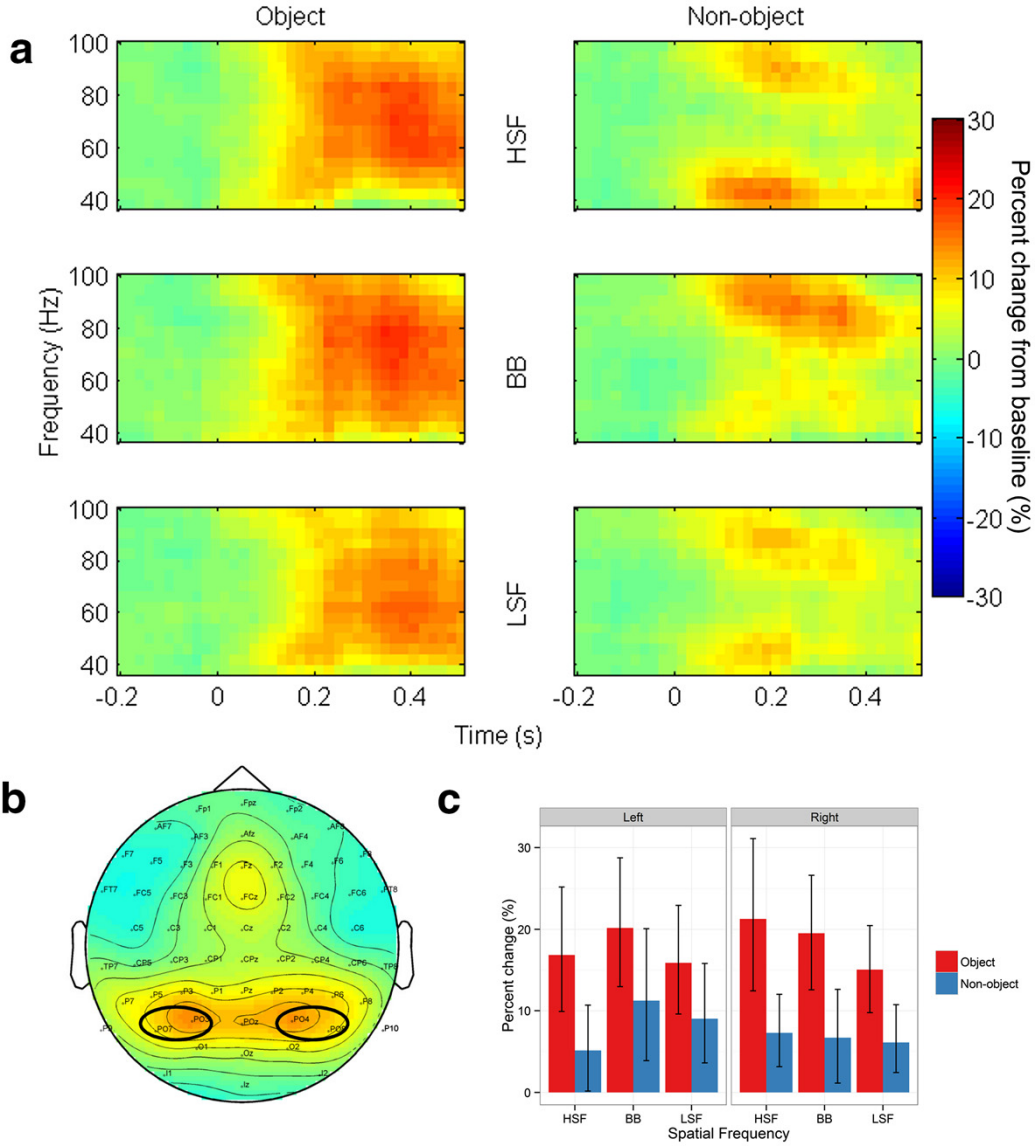
**Grand mean total gamma band activity. (a)** Mean percent change from baseline in tGBA for each individual condition. Left column shows activity on object trials, right column shows activity on non-object trials. **(b)** topography of tGBA averaged across all conditions, 40–90 Hz, and 200-500 ms. Black ovals indicate the electrode clusters used for analysis. **(c)** Bar graph showing mean percent change in tGBA for each condition. Red bars show tGBA on object trials, blue on non-object trials. Error bars depict 95% confidence intervals.

Total gamma band activity showed a significantly greater increase [*F*(1,14) = 28.3, *p* < .001, ƞ^2^_g_. = .14] relative to baseline for objects (18.1%) than for non-objects (7.6%). Neither the main effects of Spatial Frequency [*F*(2,28) = 2.44, *p* = .1, ƞ^2^_g_. = .008] nor Hemisphere [*F*(1,14) = .03, *p* = .9, ƞ^2^_g_. < .001] were significant. Importantly, the two-way interaction between Object and Spatial Frequency was not significant [*F*(2,28) = 1.36, *p* = .3, ƞ^2^_g_. = .008], see Figure 8. However, there was a significant interaction between Spatial Frequency and Hemisphere [*F*(2,28) = 5.86, *p* = .007, ƞ^2^_g_. = .01]. Although no comparisons were significant in post-hoc follow-up tests (all *p*s > .1), this interaction was likely driven by smaller gamma band responses to HSF images in the left hemisphere than the right hemisphere. Note that, although Figure 8 shows the three-way interaction between Object, Spatial Frequency, and Hemisphere, this interaction was not significant [*F*(2,28) = 0.17, *p* = .8, ƞ^2^_g_. < .001]. No other interactions were significant (all *p*s *>* .3).

## Discussion

We presented high spatial frequency (HSF), low spatial frequency (LSF), and broadband (BB) images of objects and non-objects and asked participants to categorize each image as depicting a living or non-living object, or as a non-object. Models of visual object processing in which processing of LSF precedes processing of HSF, or in which different ranges of spatial frequencies are processed via different pathways [4], would predict early differences in spatial frequency processing. As predicted, we found that the P1 was sensitive to spatial frequency; unexpectedly, we found that objects also provoked a higher amplitude response than non-objects in the P1. However, these factors did not interact. We also found effects of spatial frequency and objecthood in the N1, but, in contrast to the P1, we found that they interacted. Our peak-to-peak analysis suggests the N1 was partially moderated by the P1 since it yielded different results to our analysis N1 mean amplitudes. It revealed slight differences across hemispheres which were otherwise not apparent, but, more importantly, a clear interaction between objecthood and spatial frequency. As predicted, we found an effect of spatial frequency on evoked gamma band activity (eGBA) but no effect of objecthood. And while the overall difference between objects and non-objects in total gamma band activity (tGBA) was substantial, tGBA was not modulated by spatial frequency overall. These results are broadly consistent with a model in which different spatial frequencies are processed by different routes.

### Event-related potentials

Schendan and Lucia [43] suggested that object sensitivity in the P1 and N1 time windows largely reflects figure-ground segregation. We found that the P1 exhibited object-selective enhancement, with more positive amplitudes for objects than non-objects. Salient local contrast edges strongly contribute to figure-ground segregation. Given that they are present in object images but are largely reduced or absent in non-objects, this may drive early differences between these classes of image. A possible alternative explanation for is that these differences reflect a task difference between objects and non-objects. For non-objects, the task was finished once the decision had been made that the stimulus was a non-object, whereas further processing would have been required to establish whether objects were living or non-living. Thus, it is possible that the level of attention paid to objects was higher than to non-objects, since the task was harder for objects.

If this were correct, the same explanation might hold for N1 differences between objects and non-objects. However, in contrast to the P1, the N1 showed an interaction between objecthood and spatial frequency in our analysis of mean amplitudes. Once P1 amplitudes were taken into account with a peak-to-peak amplitude analysis, this interaction still obtained, albeit with some minor differences. In both analyses, although there were small but non-significant differences in patterns for HSF and LSF images across hemispheres, there was a clear effect of objecthood for broadband images, with a less negative N1 for BB non-objects than for any other category of image. In fact, the N1 was almost absent for BB non-objects. HSF and LSF images, on the other hand, produced similar negativities for objects and non-objects. An attention account thus seems unlikely to fully account for this pattern, since it would predict overall less negative N1 amplitudes for non-objects, rather than only for BB non-objects.

As noted earlier, task differences may alter spatial frequency processing in the N170 to faces [44]. Previously, we did not find evidence for such task-related differences in the N1 to objects [12]. In that experiment, participants switched between a basic-level, grammatical gender classification task and a superordinate, living/non-living categorization task. The visual N1 may index fine-grained discrimination processes [45], which would still be ongoing for most stimuli. For all objects, participants would likely still be determining whether the object was living or non-living. It is possible that for BB non-objects sufficient processing had been completed for little further processing to be required. BB object categorization was as fast as categorizing a BB non-object as a non-object, which was not true for LSF or HSF object categorization, suggesting an overall processing advantage for BB images. To relate the N1 findings to models such as Bar et al.’s [4], in which LSF are processed fastest and used to guide subsequent HSF processing, the BB images are the only images which provide both HSF and LSF information, and thus that have viable information to process early and inform subsequent processing. Thus, BB images should show different patterns to HSF and LSF only images, since both the initial LSF guess and subsequent HSF confirmation can proceed as usual.

In addition, the P1 was independently influenced by spatial frequency, with higher amplitudes for BB images than for HSF images, and higher amplitudes for HSF images than LSF images. Our spatial frequency effect is consistent with our previous study on object categorization at different levels of specificity [12], in which we found higher P1 amplitudes for HSF relative to LSF objects. That the P1 was greater for images containing HSF (since BB images also contain HSF) than LSF only images might suggest that the P1 largely reflects the HSF pathway. However, this would not necessarily predict that the BB images would drive a larger P1 than HSF images. One possibility is that total spectral power may have driven this difference [46]. Although we matched the images for luminance and contrast, filtering necessarily removes large regions of spectral power from an image, and thus a BB image will necessarily have higher spectral power across a broad range of frequencies than a HSF image. This may drive a stronger response than a stimulus targeted a particular range of frequencies.

### Gamma band activity

As predicted, we found an effect of spatial frequency but not of objecthood in eGBA. In a previous report, there was higher eGBA in response to low frequency stimuli compared to high frequency stimuli [21], whereas here, we found higher eGBA in response to high frequency stimuli. Fründ et al.’s difference was between responses to 1 cycle per degree (cpd) and 10 cpd stimuli; there was no significant difference between 1 cpd and 5.5 cpd stimuli, which are approximately the cut-offs used for our stimuli (~0.9 cpd and ~4.7 cpd). Note also that our stimuli are complex, and contain information at different spatial frequencies, whereas the sine-wave gratings used by Fründ et al. do not. Thus there are a number of differences in the stimuli across the studies which may make the two difficult to directly compare. Nevertheless, this does indicate that spatial frequency may interact with other factors in driving eGBA. Furthermore, that eGBA did not significantly differ for objects and non-objects, in keeping with many previous findings [15,18,19], does not support the suggestion that evoked GBA reflects the matching of a stimulus to a stored representation [47].

In contrast to the lack of an effect of objecthood on eGBA, the overall difference between objects and non-objects in tGBA was substantial, with a relatively large effect size (ƞ^2^_g_. = .14). This effect was not modulated by spatial frequency, in keeping with our suggestion that it should reflect predominantly high-level stimulus characteristics, and specifically, objecthood. There was a small but significant interaction between spatial frequency and hemisphere, which was driven by slightly lower tGBA in response to HSF stimuli in the left hemisphere. However, the pattern of previous results regarding hemispheric spatial frequency specialization is diverse and given that the effect was weak we do not make strong further interpretations [48-53]. We would suggest that this interaction indicates that ongoing, artefact-corrected tGBA may not reflect only the accessing of high-level, abstract object representation, though the data here were consistent with that being its primary role.

With respect to models of visual object processing in which LSF are processed first followed by HSF [4], our data is most consistent with tGBA only reflecting later stages of such models. tGBA activity in response to non-objects was in general also above baseline levels (see Figure 8). It thus seems unlikely that tGBA is driven by semantic information alone, given the absence of such information from our non-object stimuli. In regard to the physiological role of this gamma band activity, we would speculate that it may result from recurrent processing in higher visual cortices: it begins shortly after the time at which Bar et al.’s [4] model predicts that such processing begins in infero-temporal cortices with the back-projection of an object-category guess from OFC. As such, the onset of this activity may vary in time somewhat, dependent on the speed at which previous steps in the processing chain are completed.

## Conclusions

Overall, we found that the visual event-related P1 component showed independent modulations by objecthood and spatial frequency, while the N1 showed varying amplitudes based on a combination of both spatial frequency and objecthood. We would speculate that rather than early sensitivity to objecthood in the P1 reflecting early high-level processing, it may be related to figure-ground organization. The reduction in the N1 response to BB non-objects may reflect relatively easy categorization of these stimuli as non-objects, given that they had both high and low spatial frequency information. Thus, little further visual discrimination was necessary for such stimuli. Additionally, artefact-corrected tGBA is relatively insensitive to spatial-frequency, suggesting that it may be the activation of a high-level, relatively abstract object representation. The elevated gamma relative to baseline for non-objects is consistent with the late gamma response to some extent reflecting ongoing perceptual processing, possibly supplemented by high-level representational analysis. Thus, our ERP and gamma band results are consistent with different, low and high spatial frequency ranges being processed by different pathways at different speeds during object recognition.
